# An Ensemble Approach to Predict Early-Stage Diabetes Risk Using Machine Learning: An Empirical Study

**DOI:** 10.3390/s22145247

**Published:** 2022-07-13

**Authors:** Umm e Laila, Khalid Mahboob, Abdul Wahid Khan, Faheem Khan, Whangbo Taekeun

**Affiliations:** 1 Department of Computer Engineering, Sir Syed University of Engineering and Technology, Karachi 75300, Pakistan; ulaila@ssuet.edu.pk; 2 Department of Software Engineering, Sir Syed University of Engineering and Technology, Karachi 75300, Pakistan; kmahboob@ssuet.edu.pk; 3 Department of Computer Science, University of Science and Technology Bannu, Bannu 28100, Pakistan; wahidkn@gmail.com; 4 Department of Computer Engineering, Gachon University, Seongnam 13120, Korea

**Keywords:** data mining, diabetes dataset, prediction, ensemble techniques, AdaBoost, Bagging, Random Forest

## Abstract

Diabetes is a long-lasting disease triggered by expanded sugar levels in human blood and can affect various organs if left untreated. It contributes to heart disease, kidney issues, damaged nerves, damaged blood vessels, and blindness. Timely disease prediction can save precious lives and enable healthcare advisors to take care of the conditions. Most diabetic patients know little about the risk factors they face before diagnosis. Nowadays, hospitals deploy basic information systems, which generate vast amounts of data that cannot be converted into proper/useful information and cannot be used to support decision making for clinical purposes. There are different automated techniques available for the earlier prediction of disease. Ensemble learning is a data analysis technique that combines multiple techniques into a single optimal predictive system to evaluate bias and variation, and to improve predictions. Diabetes data, which included 17 variables, were gathered from the UCI repository of various datasets. The predictive models used in this study include AdaBoost, Bagging, and Random Forest, to compare the precision, recall, classification accuracy, and F1-score. Finally, the Random Forest Ensemble Method had the best accuracy (97%), whereas the AdaBoost and Bagging algorithms had lower accuracy, precision, recall, and F1-scores.

## 1. Introduction

Due to rising living standards, diabetes has become more prevalent in people’s everyday lives. Diabetes, commonly referred to as diabetes mellitus, is a chronic condition brought on by a rise in blood glucose levels [1,2]. Numerous physical and chemical tests can be used to detect this condition. Diabetes that is left untreated and undetected can harm vital organs including the eyes, heart, kidneys, feet, and nerves, as well as cause death [3,4].

Diabetes is a chronic condition that has the potential to devastate global health. The World Health Organization (WHO) has conducted recent studies that reveal an increase in the number and mortality of diabetic patients globally. The WHO anticipates that by 2030, diabetes will rank as the seventh leading cause of death [5,6,7]. According to data from the International Diabetes Federation (IDF), there are currently 537 million diabetics worldwide, and this figure is expected to be 643 million by 2030 [8]. 

The only method of preventing diabetes complications is to identify and treat the disease early [9]. The early detection of diabetes is important because its complications increase over time [10]. 

Diabetes prediction is important for proper treatment to avoid further complications of the disease. Numerous studies have been conducted on disease prediction, including diagnosis, prediction, categorization, and treatment. Numerous ML (machine learning) algorithms [11,12,13] have been utilized, according to a recent study, to identify and forecast diseases [14,15,16]. They have led to a notable increase in the efficiency and advancement of both conventional and ML approaches. Various machine learning algorithms and ensemble techniques have been used for the classification of diseases. However, according to the research history, none of them have been able to attain good accuracy, i.e., more than 80% [17]. R. Saxena et al. [18], in 2022, presented a full comparison of the available studies related to the diabetes prediction classification model, which identified the research gap that has been overcome in our research. The authors concluded that the dataset was subjected to general machine learning methods, with just one author (K. Hasan et al. [19]) employing the AdaBoost and gradient boost techniques. Therefore, a system that can produce more accurate findings is fast in terms of processing, and is more useful for prediction purposes must be devised. The aim of this study is to increase the accuracy of machine learning ensemble standard algorithms (including AdaBoost, Bagging, and Random Forest) by analyzing the UCI diabetes dataset and comparing their performances.

After examining the contributions made by several authors and researchers, it is evident that it is difficult to predict which attribute in the dataset plays an important role and that optimum feature selection cannot ensure significant quality, i.e., 100% accuracy. The majority of researchers employ a variety of classification techniques, including Bayesian inference, support vector machines, decision trees, random forests, k-nearest neighbors, multilayer perceptrons, and logistic regression. Few researchers have developed a technique that can accurately anticipate cases using recurrent neural networks or deep learning. A comparison of the research studies considered in this research is presented in Table 1. The distinctive attributes that address the early-stage risks of diabetes and the ensemble techniques with higher accuracy (specifically Random Forest, i.e., 97%) are the most significant physical conclusions of this investigation.

The remaining sections of the manuscript are organized as follows: In Section 2 methodology and data preprocessing are elaborated. The findings of the analysis are detailed in Section 3 followed by a discussion in Section 4 to justify the novelty of this exploration effort. Finally, Section 5 describes the conclusions of this paper.

## 2. Data Preprocessing and Methodology

Data preprocessing is a crucial stage in data mining when dealing with incomplete, noisy, or inconsistent data that transforms the data into a usable and optimal form [17,20,32]. To continually formulate data in a coherent and correct form, data preparation covers different activities such as data cleaning, data discretization, data integration, data reduction, data transformation, and so on [32]. For this case study, diabetes data with 17 attributes were collected from the UCI repository which contains different datasets. The dataset utilized here comprises 17 attributes reflecting patient and hospital outcomes. It has been used to assess the accuracy of the prediction by applying ensemble techniques and is made up of clinical treatment data that were gathered by direct surveys from Sylhet Diabetes Hospital patients in Sylhet, Bangladesh, and were validated by the doctors [33].

Some data mining techniques find discrete characteristics easier to deal with. Discrete attributes, often known as nominal attributes, are those that characterize a category. Ordinal characteristics are those qualities that characterize a category and have significance in the order of the categories [32]. Discretization is the process of turning a real-valued attribute into an ordinal attribute or bin. A discretize filter was applied here because the input values are real, and it could be useful to assemble them into bins [34].

**Figure 1:**
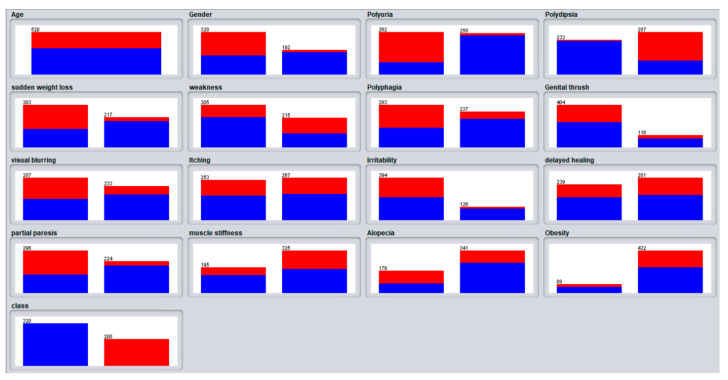
Preprocessing visualizations of the attributes.

In this study, 520 instances are used, with 17 attributes including a class attribute used to predict the positive and negative rate of chances of having diabetes or not. The list of attributes with their values is shown in Table 2 and the preprocessing results of individual attributes are shown in Figure 1.

The relevant attributes are tested in this research using the Chi-Square attributes selection technique [35]. The most important attributes establish a link between two categorical variables, specifically, a period, which is a relationship between observed and predicted frequency. For diabetic data, the Chi-Square technique is applied to calculate the attribute scores [23]. A cross-validation with 10-fold was used. This is a typical assessment approach to include the systematic division in percentages. It divides a dataset into ten sections and then tests every section separately. This yields ten assessment results that are then averaged. When conducting the first division in the “stratified” cross-validation, it makes certain that every fold has the equivalent percentage of the value of the class. For the complete dataset, the learning algorithm is repeated for the final (11th) time to produce the output after 10-fold cross-validation and hence will produce the findings of evaluation [36].

The ensemble techniques have been used on diabetes data because the number of diabetic patients is rapidly increasing and therefore it is important to pre-determine the chances of having diabetes or not in the future. Ensemble learning is a data mining approach that integrates many different techniques into a single optimum predictive model to decrease bias and variance, or enhance predictions. When compared to a single model, this technique provides a better predictive performance. For this study, AdaBoost, Bagging, and Random Forest ensemble techniques were used to predict the early stage of diabetes risk [37]. Weka was used for data exploration, statistical analysis, and data mining. Weka’s default settings were used [38]. AdaBoost is a classification problem-solving ensemble machine learning technique. It is part of the boosting family of ensemble techniques, which add new machine learning models in a sequence, with successive models attempting to correct prediction errors caused by previous models. The first effective implementation of this sort of model is AdaBoost. Short decision tree models, each with a single decision point, were used in the development of AdaBoost. Decision stumps are the common name for such short trees [35]. Bootstrap Aggregation, often known as Bagging, is an ensemble technique for regression and classification. A statistical measure such as a mean is calculated from several random samples of data using the Bootstrap approach (with replacement). When there is a limited amount of data and a more reliable estimate of a statistical quantity, this is a good approach to implement. It is a strategy that works best with models that have a low bias and high variance; by implication, their predictions are heavily reliant on the data they were trained on. Decision trees are the most often used Bagging method that meets this criterion of high variance.

Random Forest is a decision tree classification and regression technique based on Bagging. The disadvantage of bagged decision trees is that they are built using a greedy algorithm that determines the optimal split point at each phase of the tree construction process. As a consequence, the resultant trees have a similar appearance, which decreases the variance of the predictions from all the bags, lowering the robustness of the predictions [39].

## 3. Results 

AdaBoost, Bagging, and Random Forest are the three ensemble techniques used in the proposed methodology to predict the early risk of diabetes. The rate of correct classifications, either for an independent test set or utilizing some variant of the cross-validation notion, is known as classification accuracy. The kappa statistic evaluates a prediction that matches the real class with a 1.0 correlation. The Mean Absolute Error (MAE) merely assesses the average measure of errors in a group of estimates without the direction of the errors. For continuous variables, it simply evaluates accuracy. The Root Mean Squared Error (RMSE) is a quadratic notching method that primarily assesses the error’s average degree. Relative values are nothing more than ratios with no units (see Table 3).

**Figure 2:**
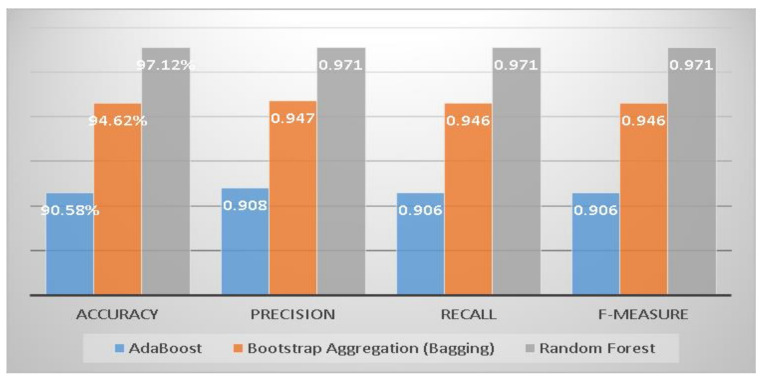
Comparison of accuracy, precision, recall, and F-measure of ensemble classifiers.

This section compares three machine learning ensemble techniques for diabetes mellitus risk classification into positive and negative groups for accuracy, precision, recall, and F-measure of all standard methods [39]. The Random Forest approach achieved the highest accuracy, precision, recall, and F-measure compared to the other two ensemble techniques (see Figure 2). 

**Figure 3:**
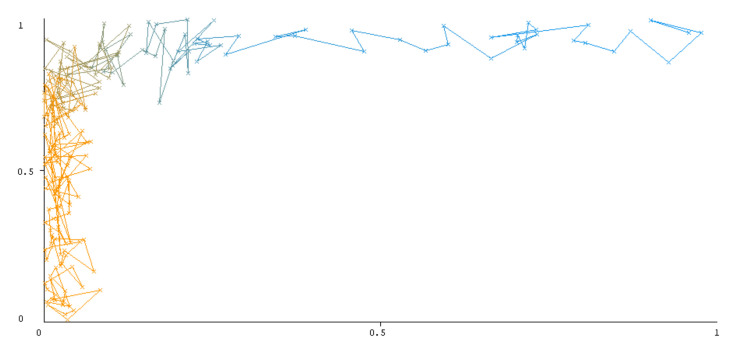
Threshold curve of a positive class.

**Figure 4:**
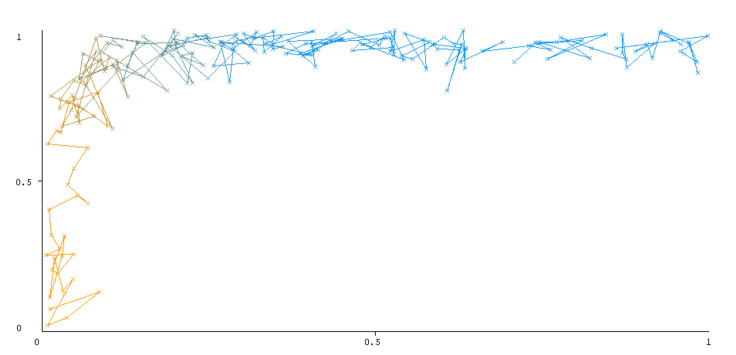
Threshold curve of a negative class using AdaBoost.

**Figure 5:**
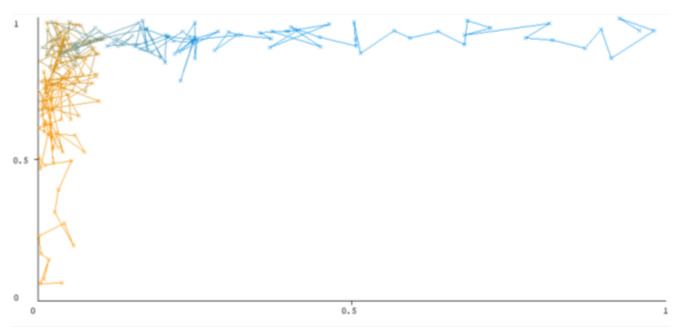
Threshold curve of a positive class using Bootstrap Aggregation (Bagging).

**Figure 6:**
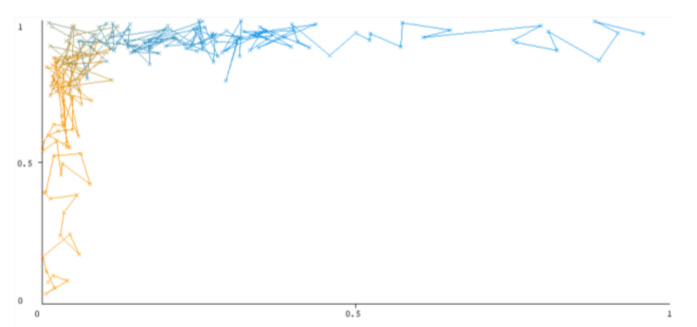
Threshold curve of a negative class using Bootstrap Aggregation (Bagging).

**Figure 7:**
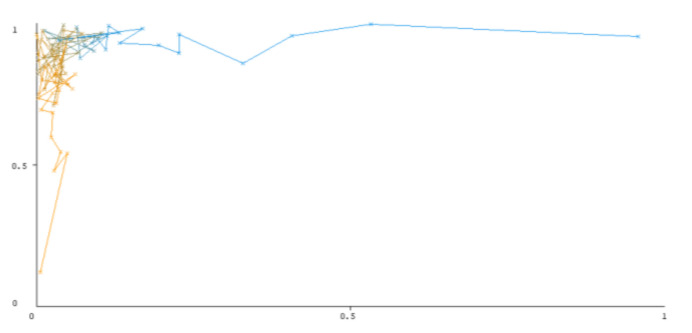
Threshold curve of a positive class using Random Forest.

**Figure 8:**
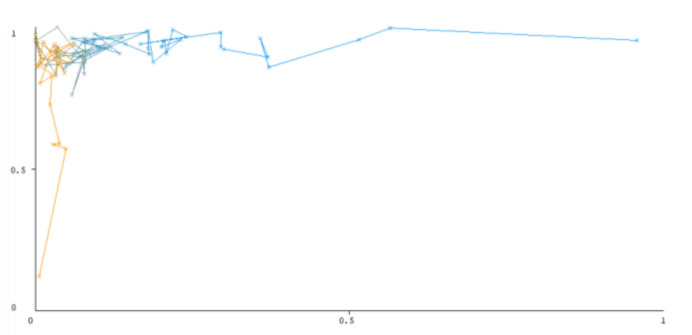
Threshold curve of a negative class using Random Forest.

The threshold curve visualizations generated from the Weka for each class, i.e., positive and negative using all three ensemble techniques, are shown in Figure 3, Figure 4, Figure 5, Figure 6, Figure 7 and Figure 8 below. It produces points that show prediction tradeoffs by changing the threshold value between classes. The common threshold value of 0.5, for example, indicates that the forecasted probability of “positive” must be more than 0.5 as “positive”. The generated dataset shows the precision/recall tradeoff or analyzes the ROC curve (true positive rate vs. false positive rate). In each scenario, Weka changes the threshold on the class probability estimations. The AUC is calculated using the Mann–Whitney statistic.

A greater performance is shown by classifiers that provide curves that are closer to the top-left corner. There are two classes in the dataset, i.e., a positive class and a negative class, which means that classifiers predict whether a person will have a risk of diabetes or not in order to identify signs at an early stage. So, ROC curves were generated using three ensemble classifiers which are visualization tools that can explain in a clinically sensitive manner whether a classifier is appropriate or not when employed for analysis. The plots of area under ROC are obtained using the AdaBoost technique with 97.9% for the positive class and 92.3% for the negative class, using the Bootstrap Aggregation (Bagging) approach, with 98.7% for the positive class and 95.3% for the negative class, and using the Random Forest technique, with 99.8% for the positive class and 99.4% for the negative class. As shown in the figures of both classes, an optimal threshold is unsubstantiated with respect to the true- and false-positive rates for each classifier (see Figure 3, Figure 4, Figure 5, Figure 6, Figure 7 and Figure 8).

**Figure 9:**
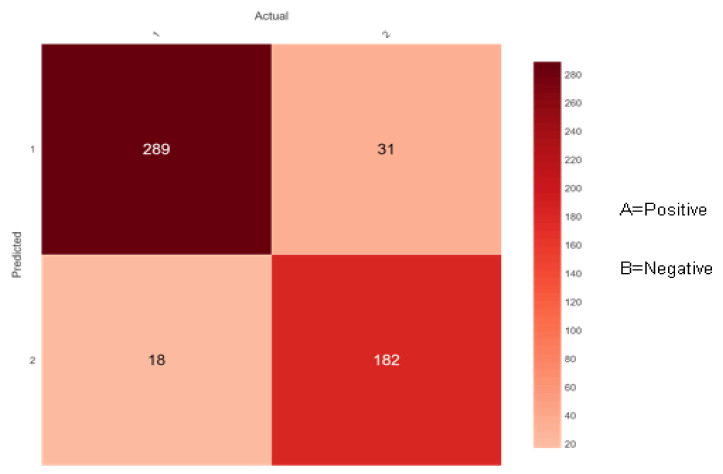
Confusion matrix of AdaBoost.

**Figure 10:**
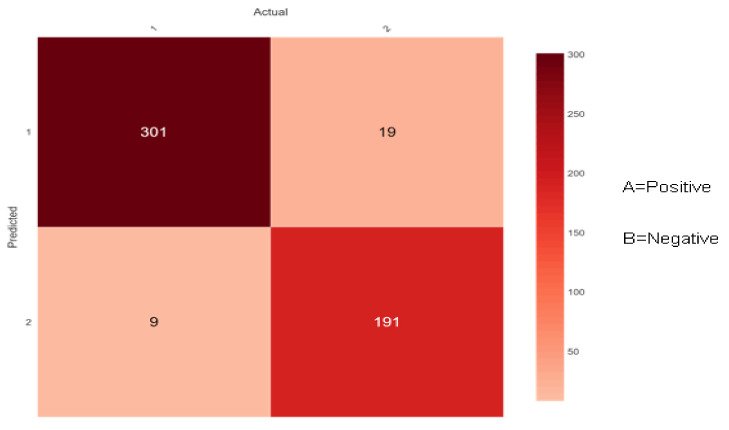
Confusion matrix of Bootstrap Aggregation (Bagging).

**Figure 11:**
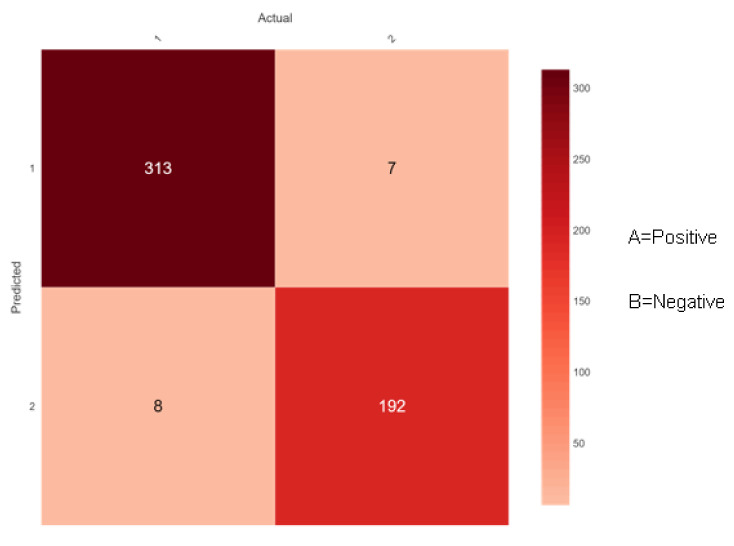
Confusion matrix of Random Forest.

The confusion matrix for diabetes risk data that the proposed ensemble classifiers properly or erroneously predict is shown in Figure 9, Figure 10 and Figure 11 below. The performance of the classifier is used for predicting the early-stage risk of diabetes and the actual values are describing the confusion matrix. 

Figure 9 shows 307 (289 + 18) instances of the actual class ‘A’ with a positive class. Here, the ensemble classifier AdaBoost predicted 289 correctly as class ‘A’ and 18 wrongly as class ‘B’. Likewise, there are 213 (31 + 182) instances of the actual class ‘B’ with a negative class. Here, the ensemble classifier AdaBoost predicted 182 correctly as class ‘B’ and 31 wrongly as class ‘A’.

Figure 10 shows 310 (301 + 9) instances of the actual class ‘A’ with a positive class. Here, the ensemble classifier Bootstrap Aggregation (Bagging) predicted 301 correctly as class ‘A’ and 9 wrongly as class ‘B’. Similarly, there are 210 (19 + 191) instances of the actual class ‘B’ with a negative class. Here, the ensemble classifier Bootstrap Aggregation (Bagging) predicted 191 correctly as class ‘B’ and 19 wrongly as class ‘A’. 

Figure 11 shows 321 (313 + 8) instances of the actual class ‘A’ with a positive class. Here, the ensemble classifier Random Forest predicted 313 correctly as class ‘A’ and 8 wrongly as class ‘B’. Similarly, there are 199 (7 + 192) instances of the actual class ‘B’ with a negative class. Here, the ensemble classifier Random Forest predicted 192 correctly as class ‘B’ and 7 wrongly as class ‘A’. Along the diagonals of the depiction, all accurate predictions are shown in dark red color.

**Figure 12:**
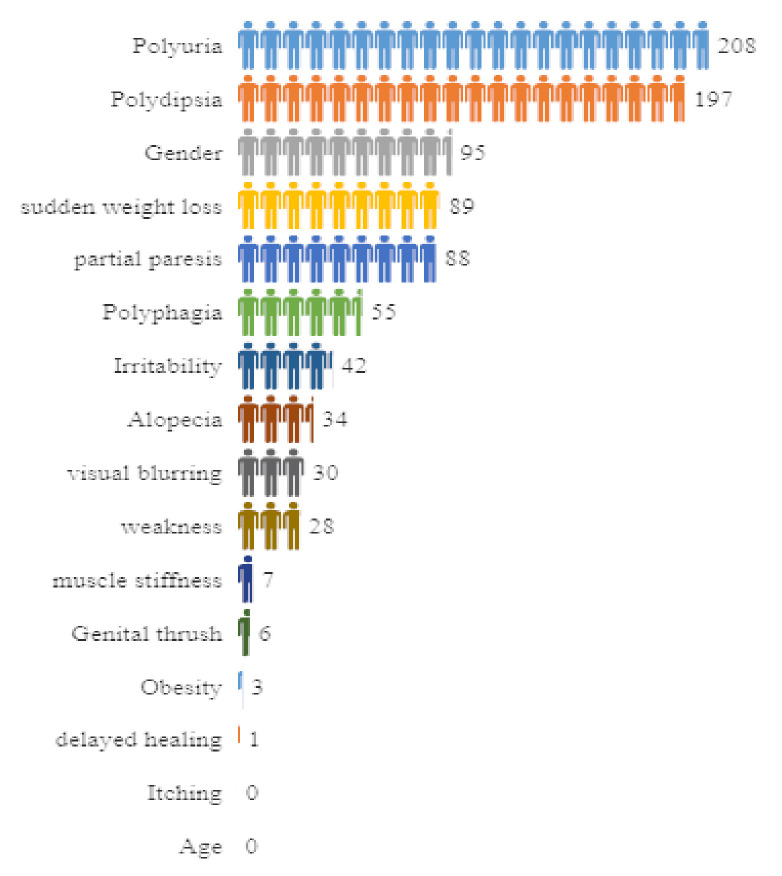
Computational representation of the attributes with their scores obtained from the Chi-Square technique.

Lastly, the Chi-Square attribute selection technique calculates the results and provides them in a format (number of attributes in a table, attributes specification, and score) applied to the diabetes data, as shown in Figure 12.

The attribute Polyuria gives the highest score, i.e., 208 approximately, whereas the attributes Age and Itching yield the lowest scores, i.e., 0. It is not necessary to extract any attributes since the Chi-Square technique discovers any attributes that do not belong to <1. It is important to note that the score obtained from the Chi-Square method indicates the attribute Polyuria (which is a syndrome in which a person urinates more frequently than usual and passes abnormally large volumes of urine each time they urinate). This is a strong indicator of diabetes risk and it contributes to the major score as well. On the other hand, the attribute Age besides Itching is associated with the lowest score; however, age is one of the highest major risk factors for diabetes, which is more common in older people, and thus can never be overlooked in analysis.

## 4. Discussion

As discussed earlier, diabetes mellitus is a long-term chronic illness that becomes more severe over time. Because the body is unable to efficiently utilize glucose, blood glucose levels rise to unhealthy levels. The hormone insulin, which regulates blood glucose levels, is not produced by the pancreas in sufficient amounts. Diabetes can cause heart disease, blindness, stroke, kidney failure, sexual dysfunction, lower-limb amputation, and difficulties during pregnancy in women if it is not treated at the proper time. The risk of acquiring diabetes is higher in those who are overweight, physically inactive or have a family history of the disease.

Therefore, it is important to identify diabetes in its early stages. Because most medical data are nonlinear, aberrant, correlation-structured, and complicated in composition, analyzing diabetic data is highly difficult. Early diabetes mellitus diagnosis necessitates a different method from previous methods. Therefore, ensemble approaches such as AdaBoost, Bagging, and Random Forest were employed in this study using a UCI dataset. The reason for using the diabetes dataset from the UCI machine learning repository in this study is that the data are cleaned and summarized by factors including attribute types, the number of instances, and the number of distinctive attributes.

From a clinical perspective, the attributes used in this study are derived from real-world experiences and they are identified as interesting and introduce the challenges of early signs and symptoms of diabetes such as polyuria which is a frequent urination condition, polydipsia which is an increased thirst condition, weakness or fatigue, and many others. However, excavating the generalizability of the model findings is a crucial subject for further exploration. The contribution and novelty of this research is that it employed Random Forest and Bagging techniques on a UCI diabetes dataset containing distinctive attributes (which has never been done before) to obtain useful insights to predict diabetes risk early.

## 5. Conclusions

Diabetes is a chronic disease that affects many individuals nowadays. As a result, the early detection of this disease is critical. This study aims to find the most accurate and efficient ensemble techniques for predicting diabetes risk in early stages using distinctive attributes. The diabetes data were gathered from the UCI repository, and they included 17 attributes. In this study, 520 instances, including a class attribute, were used to predict the positive and negative rates of possibilities of having diabetes or not having diabetes. Three ensemble techniques were applied to predict an early-stage risk of diabetes, namely AdaBoost, Bagging, and Random Forest. After applying cross-validation with 10-folds using each technique, in comparison to the other two ensemble approaches, Random Forest gives the best accuracy, precision, recall, and F-measure.

Finally, the Chi-Square attributes selection approach evaluates the relevant attributes in this study. The scores of individual attributes are calculated using the Chi-Square method. The attribute Polyuria has the greatest value (about 208), while the attributes Age and Itching have the lowest scores (nearly 0). Surprisingly, the attribute Age has the lowest value, but cannot be excluded from the study, because it is one of the most important risk factors for diabetes. The results of this study can help people and the healthcare system in managing their health.

In the future, research should focus on the advancement of algorithms in related disciplines and use more creative and effective methods to tackle existing issues, such as different deep learning models. There is a need to gather additional data (such as standard of living and picture data), enhance data collection quality, modernize the system, and create more accurate models.

## Figures and Tables

**Table 1:** Comparison of studies.

General Information								
Author/Year	Purpose	Classifier Used	Datasets	Validation Parameters	Key Findings			
Chatrati et al. [20], 2020	To forecast the presence of diabetes and hypertension	SVM, KNN, DT, LR	PIDD	ACC, scatter plot, CM, ROC curve	ACC for SVM was 75%			
Maniruzzaman et al. [21], 2020	Create a system using machine learning (ML) to anticipate diabetes patients	LR-RF combination for feature selection, NB, DT, RF, AdaBoost	National Health and Nutrition Examination Survey	ACC, AUC	ACC 94.25%			
S. Kumari et al. [17], 2021	Improve the accuracy of prediction of diabetes mellitus using a combination of machine learning techniques	NB, RF, LR	PIDD and Breast cancer dataset	ACC, Precision, Recall, F1-score, AUC	97.02% accuracy on the breast cancer dataset		79.08% accurate results on PIMA dataset	
P. Rajendra et al. [22], 2021	Create a prediction model and investigate several methods to improve performance and accuracy	LR	PIDD and Vanderbilt	Precision, Recall, F1-score	78% accuracy for Dataset 1		93% accuracy for Dataset 2	
C. Yadav et al. [23], 2021	To use a classification technique for diabetes prediction	Chi-Square for feature selection, DT, JRIP, OneR, Bagging, Boosting	UCI repository. 9 attributes	ACC, Recall, Precision, and Fi-score	ACC for Bagging ensemble methods was 98%			
Goyal et al. [24], 2021	The development of a type 2 diabetes prediction model	Using the 10-folds cross-validation approach and the ensemble method	PIDD	ACC	ACC 77.60%			
A. Prakash [25], 2021	To enhance the performance indicators for early diabetes diagnosis	J48, NB, RF, RT, SimpleCART	PIDD	ACC, computational time, Precision, FM ROC, and PRC	ACC 79.22%			
Singh Ashima et al. [26], 2021	To use an ensemble of various machine learning techniques for predicting diabetes	SVM, NN, DT, XGBoost, RF	PIDD	ACC, Sen, Spe, Gini Index, Precision, AUC, AUCH, minimum error rate, and minimum weighted coefficient	ACC 95%			
R. Saxena et al. [27], 2022	To compare several classifiers and feature selection techniques to more accurately predict diabetes	MLP, DT, KNN, RF	PIDD	Sen, Spe, ACC, and AUC	ACC for MLP77.60%	ACC for DT 76.07%	ACC for KNN 78.58%	ACC for RF 79.8%
K. Hasan [19], 2021	To put forward a robust framework for predicting diabetes	SVM, KNN, DT, MLP, NB, AdaBoost, XGBoost	PIDD	Sen, Spe, and AUC	ACC achieved was 78.9% by using AdaBoost		AUC Gradient boost 95%	
Tigga et al. [28], 2022	Various machine learning algorithms were used to predict the risk of type 2 diabetes	NB, RF	PIDD	ACC, Precision, Recall, and F1-score.	74.46% accuracy using RF on both datasets			
Jashwanth Reddy et al. [29], 2022	To create a model with the highest degree of accuracy for predicting human diabetes	SVM, KNN, LR, NB, GB, RF	PIDD	ACC, ROC, Precision, Recall, FM	ACC 80% using RF			
Jackins et al. [30], 2021	To discover a model for the diagnosis of diabetes, coronary heart disease, and cancer among the available data	NB, RF	PIDD	ACC	NB ACC 74.64%		RF ACC 74.04%	
Raghavendran et al. [31], 2022	Analyze a patient dataset to determine the probability of type 2 diabetes	LR, KNN, RF, SVM, NB, AdaBoost	PIDD	ACC, Precision, Recall, F1-Score, CM	AdaBoost performs well 95%			
Laila et al. (This study)	To increase the machine learning ensemble standard algorithms accuracy	AdaBoost, Bagging, RF	UCI repository	Precision, Recall, ACC, F1-score	RF performs well 97%			

**Table 2:** List of characteristics with their standards.

ATTRIBUTES	VALUE
Age	Numeric
Gender	Men = 328, Women = 192
Polyuria	✓ = 258, × = 262
Polydipsia	✓ = 233, × = 287
Sudden weight loss	✓ = 217, × = 303
Weakness	✓ = 305, × = 215
Polyphagia	✓ = 237, × = 283
Genital thrush	✓ = 116, × = 404
Visual blurring	✓ = 233, × = 287
Itching	✓ = 253, × = 267
Irritability	✓ = 126, × = 394
Delayed healing	✓ = 239, × = 281
Partial paresis	✓ = 224, × = 296
Toughness of muscle	✓ = 195, × = 325
Alopecia	✓ = 179, × = 341
Overweightness	✓ = 88, × = 432
Class	Positive = 320, Negative = 200

**Table 3:** Summary of stratified cross-validation performance metric.

	Accuracy	Kappa Statistic	Mean Absolute Error	Root Mean Squared Error	Relative Absolute Error
**AdaBoost**	90.576%	0.803	0.157	0.269	33.157%
**Bootstrap Aggregation (Bagging)**	94.615%	0.887	0.109	0.224	23.153%
**Random Forest**	97.115%	0.939	0.059	0.154	12.586%
**Ensemble Classifier**	**Root relative squared error**	**Precision**	**Recall**	**F-Measure**	
**AdaBoost**	55.436%	0.908	0.906	0.906	
**Bootstrap Aggregation (Bagging)**	46.219%	0.947	0.946	0.946	
**Random Forest**	31.709%	0.971	0.971	0.971	

## Abbreviations

Acronym	Explanation
ANNs	Artificial Neural Networks
AUCH	Area Under Convex Hull
AUC	Area Under the Curve
CART	Classification And Regression Tree
CM	Confusion Matrix
CNN	Convolutional Neural Network
FM	F-Measure
GB	Gradient Boosting
JRIP	Ripper
KNN	K-Nearest Neighbors
LR	Logistic Regression
ML	Machine Learning
MLP	Multilayer Perceptron
MAE	Mean Absolute Error
NB Tree	Naïve Bayes Tree
NB	Navies Bayes
NN	Neural Network
OneR	One Rule
PIDD	Pima Indian Diabetes Dataset
RAE	Relative Absolute Error
RF	Random Forest Classifier.
ROC	Receiver Operating Characteristic Curve
RMSE	Root Mean Squared Error
PRC	Precision–Recall Curve
RRSE	Root Relative Squared Error
RT	RandomTree
Sen	Sensitivity
Sep	Specificity
SVM	Support Vector Machine
UCI	University of California, Irvine
WEKA	Waikato Environment for Knowledge Analysis
